# Exploring mutation carriers’ preferences regarding onset and progression of disease predictions for adult-onset genetic neurodegenerative diseases: a qualitative interview study

**DOI:** 10.1007/s00439-025-02750-0

**Published:** 2025-05-26

**Authors:** Max J. Rensink, M. H. N. Schermer, A. Tibben, E. K. Bijlsma, S. T. de Bot, J. A. Kievit, L. L. E. Bolt

**Affiliations:** 1 Medical Ethics, Philosophy and History of Medicine, Erasmus University Medical Centre, Dr. Molewaterplein 40, Rotterdam, 3015 GD The Netherlands; 2 Department of Clinical Genetics, Leiden University Medical Center, Leiden, 2333 ZA The Netherlands; 3 Department of Neurology, Leiden University Medical Center, Leiden, 2333 ZA The Netherlands; 4 Department of Clinical Genetics, Erasmus University Medical Centre, Rotterdam, 3015 GD The Netherlands

## Abstract

Currently, new research projects aim to develop prognostic models that more accurately predict the age of onset and progression of disease for adult-onset autosomal dominant neurodegenerative diseases that lack disease-modifying treatments. While such predictions can be important for medical research and valuable in clinical practice, the perspectives of mutation carriers on receiving onset and progression predictions have not yet been explored. In this Dutch qualitative interview study, conducted between May and August 2023, the preferences, views, and concerns of 25 asymptomatic mutation carriers of Huntington’s Disease, Spinocerebellar Ataxia type 1, or Spinocerebellar Ataxia type 3 regarding onset and progression predictions were examined. Reasons for wanting to receive onset and progression predictions included life planning, preparing for the disease, informing family members, and reducing uncertainty and hypervigilance. Reasons against included concerns about negative psychological effects, the expectation of similar disease progression as family members, and a preference for receiving progression information at a later stage. Most participants were open towards disclosure of onset predictions but more hesitant regarding receiving progression information. The reasons expressed and the preferred predicted age ranges varied among age groups, and some differences in preferences were observed between the three diseases. These findings may guide the development and responsible implementation of such prognostic models and can support healthcare professionals in the counselling of mutation carriers of adult-onset genetic neurodegenerative diseases.

**Supplementary Information:**

The online version contains supplementary material available at 10.1007/s00439-025-02750-0.

## Introduction

Huntington’s Disease (HD) has been a paradigm for ethical discussions about presymptomatic genetic testing for late-onset genetic diseases without a disease-modifying treatment (Lilani 2005). Two important reasons for presymptomatic genetic testing for HD are informed life planning and reducing uncertainty about whether individuals at risk will develop disease symptoms in the future (Baig et al. 2016; Mandich et al. 2017; Tibben 2007). However, gaining certainty about carrying the mutation confronts identified mutation carriers with a new layer of uncertainty, as the exact timing of onset, severity, and rate of progression remain uncertain.

Currently, research projects aim to develop tests to predict the age of onset and progression of disease for adult-onset neurodegenerative disorders (NDDs) lacking a disease-modifying treatment, such as HD, Spinocerebellar Ataxias (SCAs), Amyotrophic Lateral Sclerosis, Frontotemporal Dementia, Parkinson’s Disease and Alzheimer’s Disease (CureQ 2022; Westeneng et al. 2018; Won et al. 2020). For instance, the CureQ project (CureQ 2022) aims to more accurately predict onset and disease progression for three autosomal dominant NDDs – HD (MIM 143100), SCA1 (MIM 164400), and SCA3 (MIM 109150) – characterised by an expanded CAG repeat in the mutated coding gene (Huntingtin gene, Ataxin-1 gene, and Ataxin-3 gene respectively) (La Spada and Taylor 2003). Only a few CAG repeats for these diseases have reduced penetrance – for example, 36 to 39 repeats in HD – creating uncertainty about disease development (Caron et al. 2020). When a higher CAG repeat is present, these NDDs show full penetrance, leading to progressive symptoms with clinical onset often between 30 and 50 years (Caron et al. 2020; Opal and Ashizawa 2017; Paulson and Shakkottai 2020). The CAG repeat length is inversely correlated with onset: longer repeats lead to earlier onset (Langbehn et al. 2010), but individual variance is significant. Median survival after a clinical HD diagnosis is 15 to 18 years (Caron et al. 2020). Clinical HD manifestations include motor symptoms like chorea, cognitive impairment leading to dementia, and behavioral and personality changes (Ghosh and Tabrizi 2018). SCA1 and SCA3 symptoms include a spinocerebellar syndrome, leading to imbalance and speech difficulties (Jacobi et al. 2015), non-ataxia signs or symptoms such as hyperreflexia and sensory symptoms (Jacobi et al. 2015; Schmitz-Hubsch et al. 2008), and cognitive impairment may also play a role (Schmitz-Hubsch et al. 2008). SCA1 generally progresses more rapidly than SCA3 (Jacobi et al. 2015; Scott et al. 2020). Mean survival after disease onset varies in both diseases. For adult-onset SCA1, survival ranges from 10 to 30 years after onset (Opal and Ashizawa 2017). For SCA3, average survival of 20 to 25 years has been reported (Kieling et al. 2007; Paulson and Shakkottai 2020).

Onset and progression predictions mark a new phase in prognostic testing. The primary aim of more precise onset and progression predictions lies in the research setting, where these predictions can be valuable in clinical trials to assess a potential disease-modifying treatment’s efficacy, and to determine the optimal time to start treatment once a disease-modifying treatment becomes available (Koval et al. 2022). For identified mutation carriers, these predictions could also offer valuable insights into when and how their disease symptoms may manifest. Onset and progression predictions could therefore have personal utility, aiding mutation carriers in making life decisions related to reproduction, relationships, education, career, and future planning. They may, however, also have untoward psychological effects, such as hypervigilance, anxiety, and increased worries about the future.

In this qualitative interview study, we explored the preferences, views, and concerns of asymptomatic mutation carriers of HD, SCA1, or SCA3 regarding disclosure of personal onset and progression predictions. Since a cure for HD and SCAs does not seem to be within close sight (Sampaio 2024), the current added value for carriers seems to lie in personal rather than clinical utility. Careful consideration regarding the implications and consequences of introducing such prognostic tests is therefore necessary. Recent trends in bioethics underscore the importance of gaining insight into the lived experiences of individuals affected by a certain disease or predisposition to inform ethical analysis (Kon 2009; Wangmo et al. 2018). Therefore, it is crucial to include the perspectives of mutation carriers, as onset and progression predictions may hold significant importance for them. Research specifically examining the perceived impact of onset predictions for adult-onset genetic NDDs without a disease-modifying treatment is lacking, while studies on progression predictions are limited (Mank et al. 2021; van Eenennaam et al. 2021). Notably, the existing research on progression predictions focused on individuals who had already received a clinical diagnosis.

To our knowledge, this is the first study on the expected impact of receiving onset and progression predictions for asymptomatic mutation carriers of adult-onset NDDs lacking a disease-modifying treatment. Previous research in HD has shown that decision-making processes in presymptomatic genetic testing are complex and extend beyond merely weighing pros and cons (Cox 2003). If onset and progression predictions become available, understanding the possible preferences, views, and concerns of identified mutation carriers could help healthcare professionals in providing genetic counselling. Therefore, our study offers valuable insights for the ethically responsible development of prognostic models for onset and progression predictions and can inform decisions on whether and how to offer such predictions in clinical practice.

## Methods

This multicentre, qualitative interview study is reported following the COREQ checklist (see Supplementary Material 1) (Tong et al. 2007).

### Study design

Dutch-speaking asymptomatic mutation carriers of HD, SCA1, or SCA3 aged at least 18 years were recruited from April to August 2023 in the Netherlands. Mutation carriers with confirmed clinical disease onset by a neurologist were excluded. We aimed to recruit individuals who received their presymptomatic genetic test results in a period between six months and five years before the interview, ensuring proper processing and recall. Due to recruitment difficulties, we extended this period to include results received after a few months up to ten years. A member of the Dutch HD patient organisation was consulted twice for advice on how to maximise cultural diversity within the participant group, which can be defined in various ways. With the term cultural diversity, we refer to differences in participants’ cultural, ethnic, and religious backgrounds. For instance, we sought to include participants with a migration background, such as mutation carriers with Turkish and Polish roots, as families from these backgrounds are represented in the Dutch clinical populations. Furthermore, we aimed to recruit individuals from two different age groups (under and over 40) based on the hypothesis that age influences the reasons for and against receiving onset and progression information, with younger participants likely focusing more on reproductive, educational, and career decisions.

Potential participants were recruited in two ways. First, healthcare professionals of the clinical genetics departments of three University Medical Centres (of Leiden, Nijmegen, and Rotterdam) and the neurology departments of two University Medical Centres (of Leiden and Maastricht) identified and approached candidates via email or telephone. Second, to increase the number of SCA1 and SCA3 participants, the Dutch Ataxia patient organisation invited members via email to join the study.

The interviewer (MR) contacted potential participants to inform them about the study, verify inclusion criteria, and schedule a one-time, in-person interview. Two potential participants cancelled, one due to time constraints and another due to personal circumstances. One participant was excluded due to confirmed clinical disease onset by their neurologist. Recruitment continued until data saturation was reached.

Interviews took place at participants’ homes or at the Erasmus MC. Participants were invited to bring their partner to assist in reflecting on their considerations, help articulate preferences, and provide support.

An interview guide was developed and refined through multiple discussions among authors (MR, MS, AT, and LB). Additionally, the interview guide was discussed separately with representatives of the Dutch HD and Ataxia patient organisations, which improved the interview guide’s comprehensibility. The interview guide was also practised with a PhD candidate of the Erasmus MC Medical Ethics section experienced in qualitative interviews.

The main topics of the interview guide included (1) family background regarding the disease, (2) the reasons for and against opting for presymptomatic genetic testing and its impact, (3) reasons for and against receiving an onset prediction and its potential impact, (4) reasons for and against receiving a progression of disease prediction and its potential impact, and (5) preferred characteristics of the prognostic test (see the interview guide in Supplementary Material 2). This paper focuses on topics 3 to 5. Topics 1 and 2 provide important background information; topic 2 has been extensively covered in the literature. Where relevant for context, information on topics 1 and 2 is included throughout the Results section.

### The interviews

The first interview was conducted by two authors (MR and LB). Subsequent interviews were conducted by one author (MR: male, PhD candidate medical ethics) with a background in medicine and philosophy, who received training in qualitative interviewing in healthcare. Interviews were audio-recorded, transcribed verbatim, and pseudonymised, and field notes were taken. After a few interviews, three authors (MR, AT, and LB) reviewed the interview guide and made slight modifications to it based on the insights from early data collection.

### Data analysis

Two authors (MR and LB) independently coded all interview transcripts. The coding framework developed from the initial interviews was expanded and adjusted during the coding of subsequent interviews. Discrepancies in interpretation were resolved through discussion to achieve consensus. NVivo version 12 software facilitated an inductive thematic analysis, identifying important themes. These were discussed by multiple authors (MR, MS, AT, and LB) in various combinations.

### Ethical approval and informed consent

The study proposal was granted a waiver, determining that it does not fall under the Dutch Medical Research Involving Human Subjects Act (WMO) (MEC-2022-0827). All participants and participating partners provided written informed consent.

## Results

### Study population

**Fig. 1:**
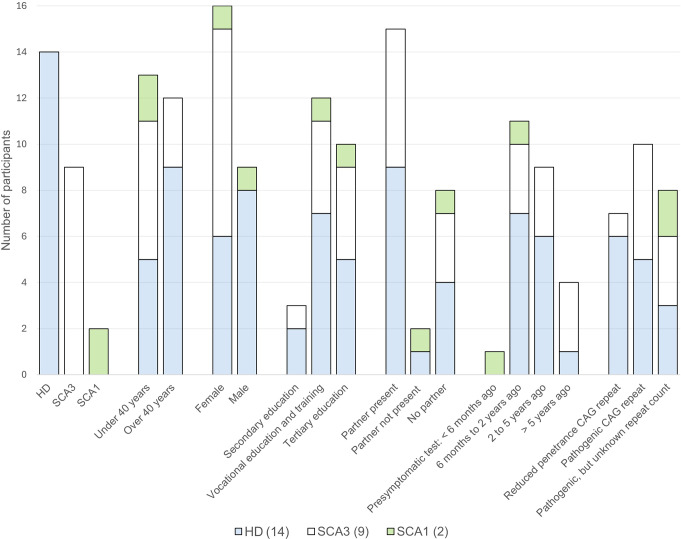
Information about study participants

We conducted 24 semi-structured interviews (lasting 32 to 83 min) with 25 participants in the Netherlands from May till August 2023. 14 HD, 2 SCA1, and 9 SCA3 mutation carriers were included and evenly distributed across two age groups (see Fig. 1). Most interviews took place at participants’ homes, with a few at the Erasmus MC (5 out of 24 interviews). 17 participants had a partner. 14 partners were present for the entire interview, while one partner joined towards the end. One participant without a partner brought a parent, who met the inclusion criteria as well. Therefore, both have been analysed as ‘participants’, leading to 25 participants across 24 interviews. For further details, see Fig. 1.

Four main themes were distinguished in the interviews, and each theme can be divided in several subthemes. The four main themes are: (1) General attitude towards receiving onset and progression information; (2) Reasons for and against receiving onset and progression information; (3) Test characteristics; and (4) Other findings. Unless otherwise specified, the presented quotes are from the participants themselves and not from their partners. Since we were only able to include two SCA1 participants, we do not specify the SCA type in the quotes to protect their privacy. Six HD participants and one SCA participant (7 out of 25 participants) had a reduced penetrance repeat, which is indicated where cited.

## General attitude towards receiving onset and progression information

Almost all HD participants and the majority of SCA participants expressed a positive attitude towards receiving (more precise) onset information. For many participants, the presymptomatic genetic test had raised new questions regarding the start of the disease.

Participant 17, SCA, under 40.

*I think at that moment [after receiving the presymptomatic genetic test result], I became a bit more aware of ‘what do I really want?’. For example*,* I don’t stick around as long at a job if I don’t like it*,* because I think*,* life is too short for that. But I also don’t live completely like*,* ‘I have to get everything out of it’*,* because I don’t feel sick and I still have fixed costs – I haven’t been told: ‘You have cancer and in six months you won’t be here anymore.’ It’s a very vague thing: on the one hand*,* you know there is something*,* that you probably won’t be able to do this and that in twenty or thirty years. But it’s uncertain*,* it could be earlier or later*,* it could be mild or severe; how do you live your life now?*

All participants reported having received information about their CAG repeat length, and nearly all mentioned its correlation with disease onset. While almost a third could not recall their exact repeat length, almost all participants knew whether their (grand)parent had a similar or different repeat length. Many used this information to estimate their own onset, although reduced penetrance carriers often emphasised the uncertainty of their test results and frequently did not attempt to estimate their own onset. Some participants contextualised their repeat length by comparing it to family members with higher repeat lengths, expressing a relative sense of relief.

Participant 27, HD, over 40.

*When I look at my mom*,* she worked until she was 64. I’m a few points below her [in repeat length]*,* so I think*,* well*,* I try to see it positively.*

Some participants mentioned receiving estimated onset information from their healthcare professional, which provided some certainty. However, many participants felt the repeat length did not provide sufficient certainty. They often highlighted the variability in onset among individuals with the same repeat length. Some participants expressed that predictions based solely on the repeat length were not useful.

Participant 6, HD, under 40.

*Participant: It’s always a gamble with the repeats. I remember a little graphic with dots*,* where certain ages [of onset] corresponded to a certain repeat length. In my case*,* you could clearly see most black dots around 45*,* from people who first experienced symptoms at that age.*

*Partner: But there were also a few around 30.*

*Participant: Around 30*,* also a few around 70.*

*Partner: And also at 50.*

A small majority of HD and SCA3 participants considered information on disease progression also to be relevant, although there was more reticence in this regard, particularly among HD participants. Especially concerning progression information, some participants stated they needed to think about it more thoroughly. Almost one third of the participants valued receiving all possible information to gain more certainty about their future. Conversely, two HD and two SCA3 participants stated they would decline all possible onset and progression information, for varying reasons (see 2.2).

Participant 16, SCA, under 40.

*[An onset prediction] makes it more real*,* and it’s just nice to*,* sometimes*,* deny it or bury your head in the sand*,* and that becomes even more difficult.*

[…]

*If I know more about the future*,* that hinders me from living in the present*,* from enjoying now. I would be preoccupied with the future and with how that looks. How will I feel then? And that makes it – if I deny it*,* I can think: ‘I had a nice day today’*,* I can live more in the present.*

## Reasons for and against receiving onset and progression information

The reasons for and against receiving onset and progression information were diverse (see Table 1). Most of the reasons discussed apply to receiving both onset and progression predictions. Hence, we address them together in this section, while also highlighting reasons specifically mentioned for one of the two.

**Table 1:** Reasons for and against receiving onset and progression information

Reasons for receiving onset and progression information	Reasons against receiving onset and progression information
Life planning *Education*,* employment*,* and work-life balance* Family planning: having (more) children* *To enjoy life to the fullest* *To plan the final stages of life*	Negative psychological effects *Confronting* *Restrains from enjoying life to the fullest* *It may lead to overlooking other diseases and health risks*
Disease preparation *To start care at the right time* *To make decisions regarding housing*	Progression information not relevant: expecting similar disease progression as in family members**
Reducing hypervigilance	Progression information not desired until later (e.g., at disease onset)**
Informing loved ones	

* Only mentioned as a reason for receiving *onset* information

** Only mentioned as a reason against receiving *progression* information

### Reasons for receiving onset and progression information

#### Life planning

Life planning emerged as a recurring theme when discussing onset and progression predictions. In many interviews, participants were positive about receiving onset and progression information because of their desire to reduce uncertainty concerning their future disease, which enables them to plan their lives more effectively. We identified four different domains of life planning.

First, receiving onset and progression information would help several participants to make decisions regarding education, employment, and work-life balance. Some participants in the younger age group stated that onset information would, for instance, aid in decisions regarding pursuing further degrees or advancing their careers. For some participants in the older age group, onset and progression information would assist in decisions about (early) retirement. Some participants expressed that onset and progression information would be valuable for determining when to start working less and spend more time with family. A few participants noted that progression information specifically would guide them in adjusting their work tasks. For example, they mentioned that, depending on the progression information, they might focus on less cognitively demanding tasks or reduce their workload on tasks that become more difficult with motor symptoms.

Participant 2, HD, under 40.

*You can prepare your employer a little bit*,* if you choose to. Look*,* you are not obliged to. But my company focuses on what you can rather than what you can’t. They will consider adjusted work activities so I can work as long as possible.*

Participant 10, HD, over 40.

*At some point*,* you want to consider certain things in your planning. I currently work full time*,* but I might choose to work three days a week at some point*,* so I can spend two days a week doing enjoyable activities.*

Second, in the younger age group, onset information could guide decisions regarding having (more) children. Some younger participants placed a high value on onset information, as they currently refrained from having (more) children due to uncertainty about disease onset. A predicted later onset in life would lead them to consider having (more) children.

Participant 4, HD, under 40, reduced penetrance.

*I wouldn’t personally consider having more children at this point. I would just skip it for now*,* because imagine if I were to have children now and it turns out to start early. My children would be fifteen years old*,* seventeen years old*,* and they would no longer have a mother.​*.

Third, many participants stated that onset and progression information would provide them with insight into when they would no longer be able to engage in activities important to them, such as travelling and spending time with family. Receiving this information would make it possible to pursue important life experiences before it is too late.

Participant 15, SCA, under 40.

*Can I still enjoy Hawaii*,* can I still surf or not? You shouldn’t put yourself in danger. I mean*,* people love snorkeling*,* diving*,* surfing; you hear what kind of sports I enjoy. And if I know that I have symptoms*,* I think it’s important to know*,* maybe I shouldn’t do that*,* maybe I can’t do that anymore.*

Lastly, some participants, especially HD participants in the older age group, expressed that onset and progression predictions would help them plan the final stages of their lives. A few participants were also concerned with making financial and legal arrangements. Some described how a family member was unable to successfully request euthanasia^1^ due to cognitive decline, emphasising the need to have plans in place before losing the ability to express their wishes. They stressed that the timing of cognitive symptom onset is very important for decisions like drafting a will.

Participant 27, HD, over 40.

*If I first experience cognitive symptoms*,* I would authorise my sister to make decisions for me. … If it starts with physical symptoms*,* I would try to find a physiotherapist. Or with chorea or – I think it [a prediction] enables you to seek more specific help.*

#### Disease preparation

Many participants expressed that receiving onset and progression information would help them prepare for the disease in a more practical way. First, it would enable some participants to start care at the right time. They highlighted the importance of starting early with interventions such as physiotherapy for the first motor symptoms or medication for emerging psychiatric symptoms. Additionally, some participants expressed concern about securing timely access to a nursing home, describing experiences with long waiting lists for their family members.

Participant 21, HD, over 40.

*I also think that is where they [the government] are heading: you should remain independent for as long as possible. But if I know*,* around that time it might start*,* I am able to say*,* for example*,* to a nursing home: ‘I want to be on the waiting list*,* because around that time a disease will start for which I’ll need extra care.’ If I know that eventually*,* when I will not be able to live independently anymore*,* I can go there*,* that would be a relief.*

Second, many participants noted that onset and progression information would assist in housing decisions. Given the prominence of motor symptoms in HD and SCAs, many participants would use onset and progression information to make home modifications, such as installing a stairlift or creating a ground-floor bedroom. Some would consider moving to a more suitable home for living with the disease. One participant and partner were in the process of buying a house but unsure about whether such considerations were relevant at this stage in life.

Participant 17, SCA, under 40.

*If you know that*,* from that moment on*,* your balance is completely gone and walking will become very difficult*,* then we know we need to create a garden where I can easily manoeuvre with a wheelchair*,* with minimal maintenance*,* and we should live on one level. Before that*,* we should do all sorts of cool trips*,* make modifications to the car*,* etcetera.*

#### Reducing hypervigilance

After receiving the presymptomatic genetic test result, many participants experienced a heightened watchfulness for potential symptoms (hypervigilance). Consequently, many participants would appreciate receiving onset and progression information, because they believed it would reduce uncertainties and thereby lessen constant symptom monitoring. One participant mentioned it would bring peace of mind to not constantly worry about disease onset after dropping something. However, another participant stated that receiving progression information specifically about the type of symptoms could also intensify hypervigilance.

Participant 16, SCA, under 40.

*I sometimes think*,* is my balance worse than before? I am concerned about that sometimes*,* while it is not yet necessary. I’m very alert on potential symptoms. If you know the kind of symptom it starts with*,* you can let it go a bit more. Although… I actually do not want to know*,* because I would focus very much on that one particular symptom.*

#### Informing loved ones

Informing loved ones also emerged as an important issue in the interviews. Participants expressed concerns about their partners, children, and other family members, wanting to provide them with information about the prospective disease. Many participants stated that knowing onset and progression is also relevant for spouses and family members, for instance to prepare for the future and to pay extra attention to potential first symptoms. Some HD participants expressed concerns about potentially overlooking these early symptoms.

Participant 5, HD, under 40.

*And for each person*,* it [the disease] is different*,* but it is good to know*,* especially for your family. They’ll have an idea of what is going to happen and how.*

Participant 21, HD, over 40.

*If I know*,* for example*,* that certain psychological symptoms start at a certain age*,* I can say to colleagues*,* friends*,* and my partner: ‘Around that time*,* you might notice something different about me*,* that I’m changing.’*

### Reasons against receiving onset and progression information

Two HD and two SCA3 participants would decline all prognostic information, often due to reasons^2^ related to the themes discussed in this section. Although the majority of the participants had a positive attitude in general towards receiving onset and progression information, many also voiced concerns and reasons against it. Some participants were concerned about potential negative psychological effects, while others mentioned they already had a perception of how the disease would manifest or were concerned about the timing of the progression information.

#### Negative psychological effects

Some participants expressed that onset and progression information could be confronting. Some of them compared onset information with a ‘ticking time bomb’ counting down until onset or feared a prediction of rapid progression. A few others viewed onset and progression information as a potential hindrance from living in the present and fully enjoying life, fearing they would become overly fixated on a prediction. For example, two SCA participants preferred not to dwell on the disease too much and to take things as they come, while an HD participant expressed fear that progression information would prevent her from engaging in enjoyable activities. Lastly, a few participants also expressed concern about neglecting other health risks due to focusing on a prediction.

Participant 29, SCA, under 40.

*My uncle passed away from cancer. So you know you have the disease [SCA]*,* you are still able to walk*,* and then*,* halfway through the timeframe of SCA*,* you hear that you have cancer. In the end*,* you don’t reach either the good or the bad scenario at all [of SCA]*,* because you have to step out of life halfway through.*

#### Expecting similar disease progression as in family members

A few participants were not interested in receiving progression information because they already had a clear idea of how the disease would manifest. They based this on observing symptom progression in family members or on acquired general progression information, and expected that their progression would mirror that of their family member(s).

#### Progression information not desired until later

In the younger age group, a few SCA participants and one HD participant voiced concerns about the timing of receiving progression information. They felt progression predictions would only be valuable just before or at the moment of clinical onset, thereby still allowing time for crucial decisions such as those regarding housing and drafting a will. At this current moment, they considered onset information most important.

Participant 13, HD, under 40, reduced penetrance.

*I think I don’t want to know right now*,* no. Because now*,* you think about*,* when will it start? I would prefer to know that instead of receiving a prediction about the progression. But the moment you know it has started*,* I would want to know: how long do I still have*,* how will it go?*

## Test characteristics

Currently, it is unclear what a test predicting onset and progression would entail, as such a test has not been developed yet. Therefore, we have asked participants about their preferences in this regard, often discussed in the context of onset prediction. This included their preferred range of onset prediction (a range of three years could result in, for example, an onset prediction between 37 and 39 years, or between 61 and 63 years) and the desired minimum reliability of the prediction.

### Preferred range of onset prediction

The preferred range of onset prediction varied among the two age groups, but not between HD, SCA1, and SCA3 participants. The majority of the participants in the younger age group would prefer a range of two to five years. Some participants within this group found a prediction more precise than two years too confronting.

Participant 4, HD, under 40, reduced penetrance.

*I think five years [range]*,* that would be great. If they were to say*,* for example*,* between 75 and 80*,* that would be great.*

*[…]*

*With that [age range of three years] I would be okay*,* but they should not tell me that it starts at my 80th or 81st. I think I would become very scared for that age.*

Participants in the older age group preferred a more precise onset prediction: all but one participant would opt for a range of one or two years. One participant’s partner suggested that ranges should become smaller as carriers get older.

Partner of participant 10, HD, over 40.

*I would almost say intervals of five years*,* but so much can change in five years that such a step is actually too large. Then you would say indeed*:* below fifty, there should be steps of five years*,* above fifty steps of three years*,* and above sixty steps of two years. That you obtain such a distribution.*

### Reliability

In the interviews, we stated that an ideal prediction would be correct 100 out of 100 times, but declared that that is difficult to reach. We subsequently asked participants what a reliability threshold regarding onset predictions would be for them. Most participants found it challenging to determine a reliability threshold for such predictions. Eventually, the vast majority settled on a minimum of 70% or higher, with only a few opting for over 90%. Many participants stressed the need for a threshold to ensure informed decision-making based on the predictions.

Participant 4, HD under 40, reduced penetrance.

*I think if you’re going to make such a prediction*,* it has to be accurate*,* because you can’t just throw out an age and then have people getting sick five years earlier*,* or ten years earlier. Then you’re really stirring up trouble*,* I think*,* because people will of course start living their lives accordingly and make important decisions about the end of life*,* or what they’re going to do with their house*,* or with their job. If it turns out that those people actually get sick five or ten years earlier and they haven’t made those decisions yet…*.

Additionally, some participants assumed that if an onset prediction is incorrect, it will still be close to the actual onset. For example, if the model predicts onset between 51 and 53 years old, some participants speculated that in case of an incorrect prediction, the actual onset might occur around 49 or 55 years old rather than significantly outside this range.

Participant 21, HD, over 40.

*70% would be the lower limit. Then you know for sure that the result of the prediction is somewhat accurate*,* that it will ultimately point in that direction. For example*,* if the prediction says: 58 years old. With 70%*,* you still know that it might be a few years below or above*,* you can still live with that. But the lower you go [in reliability]*,* the more uncertain it becomes. And then you’re actually left with mixed feelings*,* because you wanted certainty*,* but you still get uncertainty.*

## Other findings

The analysis of the interview data also brought up two other interesting topics potentially relevant for the development and implementation of a test predicting onset and progression.

### Disease onset

While some HD participants feared they might not notice the first symptoms (a known HD symptom called anosognosia (Sitek et al. 2014), relying on family, friends, or a doctor to inform them, a few SCA3 participants believed the disease had already begun. Although they reported experiencing symptoms, sometimes already for multiple years, their neurologist could not confirm whether these symptoms were linked to the disease, which is known to be difficult (Boo et al. 1998; Oosterloo et al. 2021). Their belief that the disease had already started led to a lack of interest in onset information.

Participant 14, SCA, over 40.

*There is a discrepancy: when I say that I experience symptoms*,* the medical professional says*,* ‘But I don’t see it*,* so it hasn’t started’*,* to put it bluntly. I truly feel taken seriously*,* that’s not the issue. But what is the added value in saying*,* ‘Now it’s revealing itself’? … How do you deal with that? Because I don’t believe in saying,‘That’s the moment’.​*

### Lifestyle

A few SCA and HD participants believed lifestyle factors like exercise, diet, and alcohol consumption affect disease onset and progression. Some SCA participants doubted precise onset prediction due to these factors. While all participants expressed a desire for interventions that postpone onset or delay the disease, some were specifically interested in lifestyle choices optimal for delaying or slowing down the disease, like specific (sports) activities, (vitamin) supplements, or diets.

Participant 7, SCA, over 40, reduced penetrance.

*How quickly does such a process go? What influence do alcohol*,* drugs*,* smoking*,* and obesity have on the progression? They really couldn’t say anything about that at all.*

## Discussion

In this interview study, the general attitude of most mutation carriers of HD, SCA1, and SCA3 towards receiving onset information was positive. Following their presymptomatic genetic test result, most participants desired further information about their future disease. Importantly, participants were asked to consider this information within the current context where no disease-modifying treatment is available. This study found that mutation carriers of HD, SCA1, and SCA3 often estimate their disease onset by correlating it to the observed disease onset of family members and by relating the repeat length and onset of their (grand)parent to their repeat length. This suggests a need for clarity about personal disease onset and aligns with their general willingness to receive such information.

Participants provided a wide range of reasons for wanting to receive onset and progression information. The most important reasons mirror those given by at-risk individuals for undergoing presymptomatic genetic testing, which include reducing uncertainty, life planning, and informing relatives (Baig et al. 2016; Mandich et al. 2017; Tibben 2007). Our study results suggest that presymptomatic genetic testing alone does not fully fulfil some mutation carriers’ needs in this regard, and that obtaining more precise information about disease onset and progression could provide them with meaningful benefits. Additionally, preparing for the disease and reducing hypervigilance were also noted as important reasons for seeking onset and progression information. There were some differences between the two age groups in this regard. For example, participants in the younger age group (under 40) were more likely to focus on reproductive, educational, and career decisions, whereas older participants tend to focus more on providing information to their children, retirement planning, and end-of-life decisions.

Approximately one third of the participants stated they wanted to obtain all possible onset and progression information, while four participants would decline any possible information. The other half of the participants expressed a positive attitude towards receiving onset information in general, but many were also concerned with untoward psychological effects. If implemented, it seems important to thoroughly discuss the potential consequences of receiving onset information with mutation carriers. The findings of this study can assist counsellors in discussing important reasons for and against receiving this information if it becomes available.

In general, participants were more hesitant regarding obtaining progression information, with approximately half of them unwilling to receive this information at present. Some felt this information would not significantly add to their current knowledge, as they believed their own situation would mirror that of their affected family member(s). Additionally, some participants, mainly in the younger age group, believed such information only becomes relevant later in life. This suggests that when onset and progression predictions become available, it is important to discuss their implications separately as two distinct topics.

Preferences for the range of onset predictions varied between the two age groups. The older age group preferred a more specific onset range (one to two years) compared to the younger age group (two to five years). It appears that carriers who perceive the onset as closer to the present have a stronger desire for a more precise prediction. When developing a prognostic model, it is important to consider the possibility of differing preferences among age groups. Additionally, when such a model becomes available in clinical practice, information about age group preferences can be valuable for genetic counsellors.

Furthermore, participants mentioned a wide variation in percentages regarding a reliability threshold for such a test. Many found questions about test characteristics and terms like ‘reliability’ and ‘range’ difficult to grasp. The majority of participants believed that if a prediction is incorrect, the actual onset would still occur close to the predicted range, but this remains uncertain. If this belief is not correct, it could affect their preferences regarding a reliability threshold. A test with a reliability threshold significantly below 90% as suggested by many participants could complicate the counselling process and diminish the personal utility of these predictions. Understanding percentages and probabilities in healthcare-related situations is notoriously challenging for individuals (Apter et al. 2008). Since such predictions will inevitably come with some degree of uncertainty, extensive communication about the test’s reliability should occur after its implementation. Moreover, this raises the question of what an appropriate threshold for implementation should be, as well as how and by whom it should be determined.

In addition, we only discussed one potential type of test result regarding the onset (an age range). Other strategies are also possible, such as a test that predicts whether someone will show clinical symptoms within the next x years. One participant mentioned this type of communicating such a test result. These different methods of communicating test results may be used in the counselling to ensure that the communicated results are most in line with the counselee’s preferences and needs.

Some participants had a repeat length within the reduced penetrance range, which means they currently remain uncertain about developing disease symptoms in the future. Reduced penetrance carriers often stressed the uncertainty of their test results and did not attempt to estimate their own disease onset. While being uncertain about developing symptoms in the future could potentially increase their willingness to receive onset and progression information, we did not observe a difference in willingness between reduced and full penetrance carriers regarding receiving this information. However, the sample size of this qualitative study is too small to draw definitive conclusions.

A few SCA participants wondered about whether the disease had already started, which was not confirmed by their neurologist. This raises broader questions about how disease onset will be defined in a prognostic model. For instance, such a model may be able to detect changes that occur before the current moment of clinical onset as diagnosed by neurologists. This raises questions about whether the model will be trained to predict the current moment of clinical diagnosis, or whether it will predict a different moment of onset, e.g. in a prodromal stage. For instance, in HD research, Tabrizi et al. (2022) suggested distinguishing multiple disease stages. Some of these stages are based solely on increased biomarkers, in the absence of clinical symptoms. If such a system extends to clinical practice, what will the consequences be for mutation carriers? Would they consider themselves to be ill sooner? These are important questions that should be addressed during the development and after implementation of such a model.

In general, we observed a dynamic thought process among participants. Some participants explicitly indicated that the interview questions were challenging, while others, after contemplation, arrived at different viewpoints than their initial responses. Additionally, some participants found it difficult to decide whether they would opt for a prediction, as receiving it has both benefits and drawbacks, which indicates the importance of thorough counselling. It is possible that, despite expressing their willingness to receive onset and progression information during the interview, participants might ultimately make a different decision, similar to how individuals considering undergoing presymptomatic genetic testing in the 1980s and 1990s changed their minds (Craufurd et al. 1989; Tibben et al. 1992). However, this does not weaken the relevance of our study results, as our main aim was not to determine participants’ definitive decisions regarding onset and progression predictions but to map their possible preferences, views, and concerns. In addition, many participants had observed a parent or other relatives progress through multiple stages of the disease, which shaped their understanding of their own potential future. In the literature on presymptomatic genetic testing for HD, such experiences appeared to influence some reasons for and decisions about undergoing testing (e, g, Mandich et al. (2017). While firsthand exposure to the disease’s severity may have also shaped our participants’ responses, this experience is an inherent aspect of hereditary diseases.

Our study has several limitations. First, HD, SCA1, and SCA3 have some distinct characteristics and manifestations, such as the more pronounced cognitive impairment observed in HD. We aimed to address these differences by highlighting notable differences between the diseases where relevant. Second, to capture a broad range of perspectives, we sought to include a diverse group of participants for each disease and from both age groups. However, recruiting SCA1 and SCA3 carriers proved challenging, and as a result, the Dutch Ataxia patient organisation also distributed information about the study. This may have introduced selection bias. We did not interview men with SCA3, as only women with SCA3 were willing to participate, and we were only able to include two SCA1 carriers. The age ranges varied between the diseases, with a higher proportion of HD participants being over 40 compared to those with SCA1 and SCA3. Furthermore, individuals who strongly oppose receiving any prognostic information may have been hesitant to participate. In addition, we actively sought participants from diverse cultural backgrounds but were not successful. There are several possible explanations for this. For HD, the cultural diversity within the affected population might be limited (Rawlins et al. 2016). It is, however, also likely that individuals of these groups have either not undergone presymptomatic testing or are reluctant to participate in studies, possibly due to (cultural) taboos or language barriers. Third, during the interviews, participants were encouraged to bring their partners. In our opinion, this contributed to a deeper reflection on the answers provided by the participants. However, participants might have been hesitant to fully express their thoughts in the presence of their partner. For example, some might have been afraid of the reactions of their loved ones, though there were no indications of this in the interviews. Lastly, all participants received information regarding their presymptomatic genetic test result and its implications by their genetic counsellor. However, we did not explicitly assess participants’ prior knowledge and understanding of the disease or whether they sought additional information from other sources. This potential variation in prior knowledge might have influenced their responses, although we did not observe any critical knowledge gaps during the interviews.

Future research could proceed in several directions. Firstly, it should focus on understanding the preferences and concerns of groups with different cultural and religious backgrounds within the Netherlands regarding onset and progression information. Secondly, it is important to explore the preferences, views, and concerns of healthcare professionals as well. Some authors (MR, MS, AT, and LB) are in the process of conducting an interview study with healthcare professionals (genetic counsellors, neurologists, psychosocial workers, psychologists, and specialised nurses) to map their preferences, views, and concerns regarding more precise onset and progression predictions. Ideally, that will also stimulate discussion on best practices for counselling if such prognostic models become available. Thirdly, if such models do become available, the psychological impact of more precise predictions for onset and progression should be carefully monitored.

## Conclusion

This qualitative interview study explored the preferences, views, and concerns of asymptomatic identified mutation carriers of HD, SCA1, and SCA3 regarding receiving onset and progression information. Although the primary aim of developing prognostic models predicting onset and progression is for research purposes, their implementation in clinical practice will have significant implications for mutation carriers, at-risk individuals, and their families. Therefore, the results of this study are important for facilitating the responsible development and implementation of these models. By understanding mutation carriers’ preferences, views, and concerns, researchers and healthcare professionals can better address their needs, minimise negative effects, and maximise the personal utility these models may offer.

## Electronic supplementary material

Below is the link to the electronic supplementary material.

Supplementary Material 1

Supplementary Material 2

## Data Availability

Data sharing is not applicable for this study due to privacy conditions. The interview data are personal and potentially traceable and therefore confidential.

## Footnotes

1 Under the Dutch Euthanasia Act, euthanasia is legally permitted in the Netherlands when performed by a physician who meets strict conditions, meaning that six due care criteria must be fully met (Government of the Netherlands). Regional Euthanasia Review Committees evaluate each case to ensure compliance with these criteria. These criteria include, for example, that ‘the suffering is unbearable, with no prospect of improvement’ (Government of the Netherlands). Suffering from HD, and also from SCA, can meet these criteria.

2 One participant found onset and progression information confronting and believed clarity would come naturally over time. Another expressed having everything planned out following the presymptomatic genetic test. A third participant feared losing the ability to live in the present, while a fourth participant believed the disease had already begun (see also 4.1).

## References

[CR1] Apter AJ, Paasche-Orlow MK, Remillard JT, Bennett IM, Ben-Joseph EP, Batista RM, Hyde J, Rudd RE (2008) Numeracy and communication with patients: they are counting on Us. J Gen Intern Med 23:2117–2124 18830764 10.1007/s11606-008-0803-x PMC2596505

[CR2] Baig SS, Strong M, Rosser E, Taverner NV, Glew R, Miedzybrodzka Z, Clarke A, Craufurd D, Quarrell OW (2016) 22 Years of predictive testing for Huntington’s disease: the experience of the UK Huntington’s prediction consortium. Eur J Hum Genet 24:1396–1402 27165004 10.1038/ejhg.2016.36 PMC5027682

[CR3] Boo GD, Tibben A, Hermans J, Maat A, Roos RAC (1998) Subtle involuntary movements are not reliable indicators of incipient Huntington’s disease. Mov Disorders: Official J Mov Disorder Soc 13:96–99 10.1002/mds.870130120 9452333

[CR4] Caron NS, Wright GEB, Hayden MR (2020) Huntington disease. GeneReviews^®^[Internet]

[CR5] Cox SM (2003) Stories in decisions: how at-risk individuals decide to request predictive testing for huntington disease. Qualitative Sociol 26:257–280

[CR6] Craufurd D, Dodge A, Kerzin-Storrar L, Harris R (1989) Uptake of presymptomatic predictive testing for Huntington’s disease. Lancet 2:603–605 2570293 10.1016/s0140-6736(89)90722-8

[CR7] CureQ (2022) CureQ website. Accessed 07-02-2025. https://cureq.nl/cureq-en/

[CR8] Ghosh R, Tabrizi SJ (2018) Huntington disease. Handb Clin Neurol 147:255–278 29325616 10.1016/B978-0-444-63233-3.00017-8

[CR9] Government of the Netherlands Is euthanasia allowed? Ministry of Health, Welfare and Sport. https://www.government.nl/topics/euthanasia/is-euthanasia-allowed. Accessed 10-30-2024

[CR10] Jacobi H, du Montcel ST, Bauer P, Giunti P, Cook A, Labrum R, Parkinson MH, Durr A, Brice A, Charles P (2015) Long-term disease progression in spinocerebellar ataxia types 1, 2, 3, and 6: a longitudinal cohort study. Lancet Neurol 14:1101–1108 26377379 10.1016/S1474-4422(15)00202-1

[CR11] Kieling C, Prestes PR, Saraiva-Pereira ML, Jardim LB (2007) Survival estimates for patients with Machado-Joseph disease (SCA3). Clin Genet 72:543–545 17894834 10.1111/j.1399-0004.2007.00910.x

[CR12] Kon AA (2009) The role of empirical research in bioethics. Am J Bioeth 9:59–65. 10.1080/15265160902874320 19998120 10.1080/15265160902874320 PMC2826359

[CR13] Koval I, Dighiero-Brecht T, Tobin AJ, Tabrizi SJ, Scahill RI, Tezenas du Montcel S, Durrleman S, Durr A (2022) Forecasting individual progression trajectories in huntington disease enables more powered clinical trials. Sci Rep 12:18928 36344508 10.1038/s41598-022-18848-8 PMC9640581

[CR14] La Spada AR, Taylor JP (2003) Polyglutamines placed into context. Neuron 38:681–684 12797953 10.1016/s0896-6273(03)00328-3

[CR15] Langbehn DR, Hayden MR, Paulsen JS, the P-HDIotHSG (2010) CAG-repeat length and the age of onset in huntington disease (HD): a review and validation study of statistical approaches. Am J Med Genet B Neuropsychiatr Genet 153B:397–408 19548255 10.1002/ajmg.b.30992 PMC3048807

[CR16] Lilani A (2005) Ethical issues and policy analysis for genetic testing: Huntington’s disease as a paradigm for diseases with a late onset. Hum Reprod Genetic Ethics 11:28–34 10.1179/hrge.11.2.e35336gt18776032 16270448

[CR17] Mandich P, Lamp M, Gotta F, Gulli R, Iacometti A, Marchese R, Bellone E, Abbruzzese G, Ferrandes G (2017) 1993–2014: two decades of predictive testing for Huntington’s disease at the medical genetics unit of the university of Genoa. Mol Genet Genom Med 5:473–480 10.1002/mgg3.238 PMC5606876 28944231

[CR18] Mank A, van Maurik IS, Bakker ED, van de Glind EMM, Jönsson L, Kramberger MG, Novak P, Diaz A, Gove D, Scheltens P (2021) Identifying relevant outcomes in the progression of Alzheimer’s disease; what do patients and care partners want to know about prognosis? Alzheimer’s & Dementia: Translational Research & Clinical Interventions 7: e12189 10.1002/trc2.12189 PMC8377775 34458555

[CR19] Oosterloo M, de Greef BTA, Bijlsma EK, Durr A, Tabrizi SJ, Estevez-Fraga C, de Die‐Smulders CEM, Roos RAC (2021) Disease onset in Huntington’s disease: when is the conversion? Mov Disorders Clin Pract 8:352–360 10.1002/mdc3.13148 PMC8015887 33816663

[CR20] Opal P, Ashizawa T (2017) Spinocerebellar ataxia type 1. GeneReviews^®^[Internet]

[CR21] Paulson H, Shakkottai V (2020) Spinocerebellar ataxia type 3. GeneReviews^®^[Internet]

[CR22] Rawlins MD, Wexler NS, Wexler AR, Tabrizi SJ, Douglas I, Evans SJW, Smeeth L (2016) The prevalence of Huntington’s disease. Neuroepidemiology 46:144–153 26824438 10.1159/000443738

[CR23] Sampaio C (2024) Huntington disease–Update on ongoing therapeutic developments and a look toward the future. Parkinsonism & Related Disorders: 106049 10.1016/j.parkreldis.2024.106049 38418319

[CR24] Schmitz-Hubsch T, Coudert M, Bauer P, Giunti P, Globas C, Baliko L, Filla A, Mariotti C, Rakowicz M, Charles P (2008) Spinocerebellar ataxia types 1, 2, 3, and 6: disease severity and nonataxia symptoms. Neurology 71:982–989 18685131 10.1212/01.wnl.0000325057.33666.72

[CR25] Scott SSO, Pedroso JL, Barsottini OGP, França-Junior MC, Braga-Neto P (2020) Natural history and epidemiology of the spinocerebellar ataxias: insights from the first description to nowadays. J Neurol Sci 417:117082. 10.1016/j.jns.2020.117082 32791425 10.1016/j.jns.2020.117082

[CR26] Sitek EJ, Thompson JC, Craufurd D, Snowden JS (2014) Unawareness of deficits in Huntington’s disease. J Huntington’s Disease 3:125–135 25062855 10.3233/JHD-140109

[CR27] Tabrizi SS, Gantman EC, Mansbach A, Borowsky B, Konstantinova P, Mestre TA, Panagoulias J, Ross CA, Zauderer M, Mullin AP, Romero K, Sivakumaran S, Turner EC, Long JD, Sampaio C (2022) Huntington’s Disease Regulatory Science C A biological classification of Huntington’s disease: the Integrated Staging System. Lancet Neurol 21:632–644 10.1016/S1474-4422(22)00120-X 35716693

[CR28] Tibben A (2007) Predictive testing for Huntington’s disease. Brain Res Bull 72:165–171 17352941 10.1016/j.brainresbull.2006.10.023

[CR29] Tibben A, Niermeijer MF, Roos RAC, Van De Vlis MV, Frets PG, van Ommen GJB, van de Kamp JJP, Verhage F (1992) Understanding the low uptake of presymptomatic DNA testing for Huntington’s disease. Lancet 340:8832. 10.1016/0140-6736(92)92610-r 1360126

[CR30] Tong A, Sainsbury P, Craig J (2007) Consolidated criteria for reporting qualitative research (COREQ): a 32-item checklist for interviews and focus groups. Int J Qual Health Care 19:349–357. 10.1093/intqhc/mzm042 17872937 10.1093/intqhc/mzm042

[CR31] van Eenennaam RM, Koppenol LS, Kruithof WJ, Kruitwagen-van Reenen ET, Pieters S, van Es MA, van den Berg LH, Visser-Meily JMA, Beelen A (2021) Discussing personalized prognosis empowers patients with amyotrophic lateral sclerosis to regain control over their future: a qualitative study. Brain Sci 11:1597 34942899 10.3390/brainsci11121597 PMC8699408

[CR32] Wangmo T, Hauri S, Gennet E, Anane-Sarpong E, Provoost V, Elger BS (2018) An update on the empirical turn in bioethics: analysis of empirical research in nine bioethics journals. BMC Med Ethics 19:6. 10.1186/s12910-018-0246-9 29415709 10.1186/s12910-018-0246-9 PMC5803920

[CR33] Westeneng H-J, Debray TPA, Visser AE, van Eijk RPA, Rooney JPK, Calvo A, Martin S, McDermott CJ, Thompson AG, Pinto S (2018) Prognosis for patients with amyotrophic lateral sclerosis: development and validation of a personalised prediction model. Lancet Neurol 17:423–433 29598923 10.1016/S1474-4422(18)30089-9

[CR34] Won JH, Kim M, Youn J, Park H (2020) Prediction of age at onset in Parkinson’s disease using objective specific neuroimaging genetics based on a sparse canonical correlation analysis. Sci Rep 10:11662. 10.1038/s41598-020-68301-x[pii] 32669683 10.1038/s41598-020-68301-x PMC7363828

