# Supplementary material for: Exploring mutation carriers’ preferences regarding onset and progression of disease predictions for adult-onset genetic neurodegenerative diseases: a qualitative interview study
Source: Hum Genet. 2025 May 26;144(6):665–77. doi: 10.1007/s00439-025-02750-0 (PMC12170675; doi:10.1007/s00439-025-02750-0)
Supplement: Supplementary file 2 — Supplementary Material 2 [file 439_2025_2750_MOESM2_ESM.docx]

**Supplementary Material 2: semi-structured interview guide** (translated to English)

*The interviews were conducted in Dutch. Questions in Italic were asked only when applicable. The subquestions were posed only if they were deemed relevant and if the participant had not yet addressed the specific question.*

**1. Background**

- Education, work, age, partner
- Family tree (grandparents, parents, siblings, children)
- How old were you when you became aware of the disease in the family and your own risk of the disease? How was it discussed in the family at that time? (taboo, open atmosphere, …)
  - *At what age did your parent show symptoms of the disease?*

**2. Considerations for opting for the presymptomatic genetic test**

You eventually decided to undergo testing for HD/SCA.

- How long ago was that? How old were you then? In which centre?
- How long did you think about it? (duration of the process)
- Can you tell something about your considerations for undergoing genetic testing for HD/SCA?
  - What were important reasons not to do it? Were there reasons that prevented you from getting tested?
  - *Question to partner: you have a very different position in the process. Did you consider it important to know the test result for yourself? Can you elaborate on that?*
  - *How did you feel about your partner making that decision? Could you agree with it?*
- What consequences did the result of the genetic test have?
  - On life choices (life planning, education/job choices, relationships, well-being, as a person). *Group* *16-40 years: on family planning?*
  - *Question to partner: what consequences did the test result have for you?*
- Did the genetic test for HD/SCA provide what you expected? Did the genetic test bring you something you did not expect?
- If we go back to the moment of the presymptomatic genetic test result: what did they tell you?
  - Did they tell you when they expect the first symptoms to appear?

*If the number of repeats was mentioned:*

- *What do you think your repeat length means?*
- *How did you learn about your repeat length ? Did you ask for it? Was it offered to you?*
- *What was the reason you wanted to know this?*
- *What did the genetic counsellor say about repeat lengths?*

**3. Onset and progression information**

When you received the genetic test result, you learned that you have the predisposition to develop disease symptoms. I would now like to do a thought experiment where you consider predictive information that is not yet available.

- Imagine we could precisely determine when you will develop the disease. Would you want to know?
- What consequences would this information have for you?
  - *For your well-being, important life choices (life planning, education/job choices). Group 16-40 years: for family planning?*
  - *Question to partner: how would this be for you?*
- How precise would a prediction about when the symptoms begin need to be in order to be valuable to you? Why?
  - For example: the disease begins when you are between … and … years old.
  - *Question to partner: and for you?*
- Suppose it were possible to predict with what type of symptoms the disease would start with. Would you want to know what type of symptoms the disease would start with? Why?
  - *What type of symptoms would you want to know about? (Movement, dementia, personality?) (For HD: movement control, memory issues, irritability. For SCA: symptoms like difficulty swallowing and speaking, movement control, potential cognitive symptoms such as memory problems).*
- Suppose it were possible to predict how the disease symptoms will progress, so the speed of the process. Would you want to know that? Why?
- What consequences would this information have for you?
  - *For your well-being, important life choices (life planning, education/job choices). Group 16-40 years: for family planning?*
  - *Question to partner: how would this be for you?*
- *If the model could predict it, would you want to know how long you have left to live?*
- If a doctor would perform a predictive test, the doctor ideally aims to provide 100% certainty about the result: 100 out of 100 predictions are correct. But what if such a prediction is 98/100 times certain?
  - 95%? 90%? What would be the minimum acceptable reliability?
  - *Question to partner: and for you?*

We have discussed predictions about the age at which the first symptoms will appear, the type of symptoms, and the speed of the disease process. Which of these predictions would be most important for you to know?

- Would you want to know that right now?

**Closing**

- Do you have any additions? Is there anything else you would like to mention?
- Could we contact you again if any further questions arise?
